# The Gut Microbiota: How Does It Influence the Development and Progression of Liver Diseases

**DOI:** 10.3390/biomedicines8110501

**Published:** 2020-11-16

**Authors:** Paulraj Kanmani, Kanmani Suganya, Hojun Kim

**Affiliations:** 1 Department of Rehabilitation Medicine of Korean Medicine, Dongguk University, Goyang 10326, Korea; kanmanibiotech2007@gmail.com; 2 Department of Neuropsychiatry of Korean Medicine, Dongguk University, Goyang 10326, Korea; sonymicro85@gmail.com

**Keywords:** gut–liver axis, gut-microbiota, gut dysbiosis, liver disease, lipopolysaccharide, short-chain fatty acids

## Abstract

The gut–liver axis plays important roles in both the maintenance of a healthy liver and the pathogenesis of liver diseases, where the gut microbiota acts as a major determinant of this relationship. Gut bacteria-derived metabolites and cellular components are key molecules that affect the function of the liver and modulate the pathology of liver diseases. Accumulating evidence showed that gut microbiota produces a myriad of molecules, including lipopolysaccharide, lipoteichoic acid, peptidoglycan, and DNA, as well as short-chain fatty acids, bile acids, trimethylamine, and indole derivatives. The translocation of these components to the liver exerts beneficial or pathogenic effects by interacting with liver immune cells. This is a bidirectional relationship. Therefore, the existence of crosstalk between the gut and liver and its implications on host health and diseases are essential for the etiology and treatment of diseases. Several mechanisms have been proposed for the pathogenesis of liver diseases, but still, the mechanisms behind the pathogenic role of gut-derived components on liver pathogenesis remain elusive and not understandable. This review discusses the current progress on the gut microbiota and its components in terms of the progression of liver diseases, and in turn, how liver diseases indirectly affect the intestinal function and induce intestinal inflammation. Moreover, this paper highlights the current therapeutic and preventive strategies used to restore the gut microbiota composition and improve host health.

## 1. Introduction

The gut contains diverse microbial communities that have much more genetic material than the total human genome. The gut-residing bacteria have many health beneficial effects on the host by helping the gut to produce several metabolites, hormones, and peptides. The gut microbiota itself also produces several enzymes, metabolites, and cellular components that affect the health and diseases. The anatomy of the gut has a very close relationship with the liver through the portal vein. Therefore, the gut-derived components and immune signals are transferred to the liver, where they play a role in improving and maintaining the liver functions and healthy liver [1,2]. The liver is the largest gland with a remarkable function on the host by recruiting and activating the immune cells in response to gut-derived signals and components [3,4]. The liver produces primary bile acids that alter the gut microbial composition [5]. The interaction between the gut and the liver is bidirectional. Disruptions of this interaction result in the development of several liver diseases, including hepatic inflammation, alcoholic liver disease (ALD), non-alcoholic fatty liver disease (NAFLD), non-alcoholic steatohepatitis (NASH), fibrosis, cirrhosis, and hepatocellular carcinoma (HCC) [3,6,7]. Several therapeutic approaches have attempted to improve health by restoring the gut microbiota composition, production of metabolites, and modulation of immune signaling. Therefore, this paper provides an overview of the recent updates of the gut microbiota and its interactions with the host in health and diseases. In addition, we will discuss the pathogenic association between gut microbiota and liver diseases (gut–liver axis), touching on alcoholic and non-alcoholic fatty liver diseases and virus-induced hepatitis in humans and animals. Probiotics/prebiotics and herbal medicine-based therapeutic interventions that target the gut–liver axis to improve liver diseases are also reviewed.

## 2. Gut Microbiota and Health

**Figure 1:**
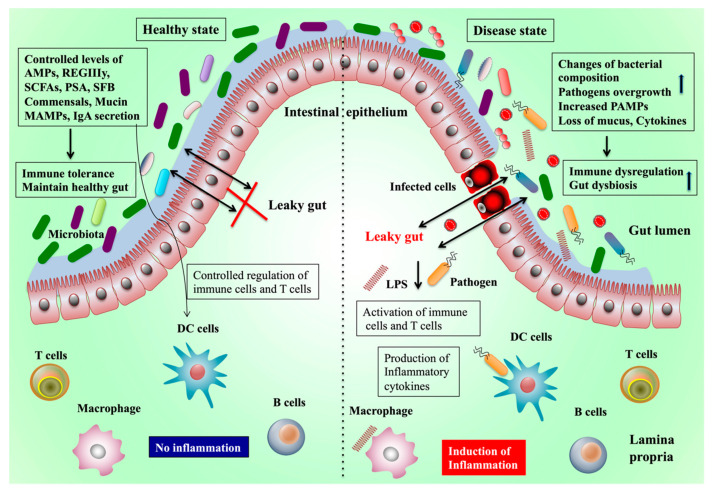
Host–gut microbiota interaction on the maintenance of a healthy gut and the induction of inflammation. The gut microbiota and its cellular components and metabolites induce immune cells, goblet, and paneth cells to produce functionally active components, immunoglobulin, and anti-inflammatory cytokines to maintain gut homeostasis and a healthy gut, with no leaky gut or inflammation. On the other hand, antibiotics, inflammatory bowel diseases (IBD), obesity, dietary modification, and other environmental factors induce gut dysbiosis, which increases the loss of gut hemostasis, impaired gut barrier integrity, and bacterial overgrowth. The disruption of the gut barrier integrity increases the permeability of pathogens and pathogen-associated molecular patterns (PAMPs) from the gut lumen to the lamina propria, where they interact with the respective toll-like receptors (TLRs) and other receptors on immune cells to dysregulate the host immunity and induce intestinal inflammation.

Gut-residing microbiota evolved with humans and became an essential organ in humans to influence health. In a healthy state, the gut microbiota has various beneficial effects in several ways (Figure 1). Four major phyla dominate in the GI: *Bacteroidetes*, *Firmicutes*, *Proteobacteria*, and *Actinobacteria*. Among them, *Firmicutes* and *Bacteroidetes* are the leading types followed by *Proteobacteria*, *Actinobacteria*, and a minor proportion of *Verrucomicrobia* and Fusobacteria phyla [8,9]. This bacterial colonization in the gut has a symbiotic relationship with the host via complex networks of interactions and crosstalk with each other. The interactions or crosstalk between the gut and liver is mainly through the gut microbiota-associated molecular patterns and their metabolic products that link the gut microbiota with other body organs system by acting as signaling molecules with immune regulatory functions.

Abbreviations used in the figure are: short-chain fatty acids (SCFA), antimicrobial peptides (AMPs), microbe-associated molecular patterns (MAMPs), pathogen-associated molecular patterns (PAMPs), inflammatory bowel diseases (IBD), regenerating islet-derived protein 3 γ (RegIIIγ), toll-like receptors (TLRs), segmented filamentous bacteria (SFB), and polysaccharide A (PSA). 

### 2.1. Production of Short-Chain Fatty Acids and Their Effects on Health

The gut microbiota produces thousands of gastrointestinal enzymes, including propionate and acetate-CoA transferase, butyrate kinase, and propionaldehyde dehydrates, which convert complex or indigestible carbohydrates of dietary food into host absorbable short-chain fatty acid (SCFAs), principally acetic acid, propionic acid, and butyric acid [10,11]. *Bacteroidetes* produce the acetate and propionate. They are absorbed immediately into the portal circulation and are transferred to the peripheral tissues, including the liver and adipose tissue, where they can be used for lipogenesis and gluconeogenesis [12,13]. Propionate has also been shown to regulate appetite, food intake, and adiposity in adults [14]. In addition, it improves the beta-cell function and stimulates insulin secretion in human islet cells [15], and modulates hepatic lipid accretion in adults with non-alcoholic fatty liver disease [16].

On the other hand, butyrate is produced by *Firmicutes*, especially *Eubacteria spp.*, and *Roseburia spp*. The bacteria-produced butyrate is utilized by colonocytes as their energy source through β-oxidation using circulating O_2_ and maintains colonic health [17]. The butyrate-producing microbiota regulates energy metabolism, ATP synthesis, and autophagy in the colon [18]. Microbe (*Lactobacillaceae* and *Ruminococcaceae*)-derived butyrate maintains cell apoptosis/proliferation by decreasing the pro-inflammatory cytokines and regulating the jejunal homeostasis in weaning piglets [19]. Butyrate has also been reported to regulate the host immune response by increasing the peripheral and colonic regulatory T (Treg) cell populations in vitro and in vivo mice [20,21]. The elevated level of butyrate also induces the level of IL-10 in the intestinal tissue of mice and maintains the balance between Treg, cytotoxic CD8+ T cells, and T_H_1 cells [21]. Bacterial butyrate production can affect the consumption of O_2_ by intestinal epithelial cells (IECs), stabilize hypoxia-inducible factor (HIF), and improve the gut barrier function [22]. Butyrate activates AMPK, which in turn promotes the reassembly of tight junctions by inhibiting the phosphorylation of myosin light chain kinase (MLCK)/myosin II regulatory light chain (MLC2) and up-regulating the phosphorylation of protein kinase C β2 (PKCβ2) at Ser660 [23]. While most SCFA present in the colon play a major role in the amelioration of intestinal inflammation, regulation of glucose homeostasis, gut motility, and suppress abnormal proliferation of colonic epithelial cells [24,25,26]. The SCFA also assists in the activation and differentiation of B cells and the production of IgM and IgA antibodies [27]. SCFA alleviates the stress-induced alternations in the brain–gut axis [28]. The elevated level of SCFA in the circulation of high-fiber diet-fed mice exhibited protection against allergic inflammation in the lung by increasing the generation of dendritic cell (DC) precursors and macrophages [29]. The beneficial effects of SCFA on the host health occur mainly through the stimulation of gut-derived hormones, such as peptide YY (PYY), glucagon-like peptide-1 (GLP-1), and intestinal gluconeogenesis [30,31]. The SCFA can also inhibit the activity of histone deacetylases (HDACs) and induce apoptosis and cell cycle arrest in colonic cancerous cells [32]. Bacterial metabolite-deficient germ-free mice showed shorter intestinal Muc2 mucin O-glycans, which are associated with the reduced expression of glycosayltransferase by intestinal epithelial cells [33]. These observations highlight the importance of microbe-derived SCFA in host health. 

The protective effects of SCFA on the host health are achieved via interactions with multiple signaling molecules or receptors. Therefore, SCFA acts as a ligand for several receptors expressed by intestinal endocrine (EE) cells, adipose tissues, enteric neurons, and other immune cells [34,35]. SCFA mediates the signal by stimulating orphan G protein-coupled receptors (GRP41, GRP43, also known as free fatty acid receptors or FFAR3 and FFAR2, and GRP109A) and inhibiting histone deacetylases or HDACs [36,37]. GRP41 and GRP43 can bind with acetate, propionate, and butyrate, but GRP43 has high affinity to short-chain fatty acids, such as acetate and propionate [38]. FFAR3 has a greater binding capability to propionate and butyrate than acetate, while, among SCFAs, only butyrate can bind and activate GPR109A [32]. The administration of SCFAs mediates protective immunity and induces an inflammatory response in mice by activating the GRP41, GRP43, ERK1, and mitogen-activated proteins kinase (MAPK) pathways [39]. An animal study reported that butyrate protects mice from colitis by increasing the differentiation of Treg cells through an interaction with the FFAR2 receptor [21]. The HDACs (HDAC1 and HDAC3) inhibition increased the frequency and function of Treg cells [40], and reduced the activation of the nuclear factor (NF)-kappa B (NF-κB) pathway and pro-inflammatory cytokines production, resulting in an improvement of host health [41]. The SCFAs treatment controlled the pathology by increasing the expression of Foxp3+, IL-17A+, and Foxp3+IL-17A+ double-positive (Treg17) cells in the oral draining LN in mice [42]. In a GPR43-dependent manner, SCFA increases the levels of regenerating islet-derived proteins III γ (RegIIIγ) and β-defensins 1, 3, and 4 by activating rapamycin (mTOR) and single transducers and activator of transcription 3 (STAT3) in mice [43]. Intriguingly, Park et al. [44] reported that SCFAs directly induced the differentiation of naïve T-cell into TH1, TH17, and IL-10+ T cells without interacting with the GPR41 and GPR43 receptors. SCFAs exhibit their regulatory functions via inhibition of HDACs and regulation of mTOM and p70 S6 kinase (S6K) pathways [44]. A recent study, however, showed that the bacterial butyrate treatment promoted TH1 cell development by inducing a higher level of IFN-γ and T-bet production, and inhibition of HDAC. On the other hand, it affected TH17 cell development by decreasing IL-17, Rorα, and Rorγt production in mice [45]. Furthermore, a butyrate treatment induced B-lymphocyte-induced maturation protein-1 (Blimp-1), resulting in higher levels of IL-10 producing T cells. Similarly, SCFAs activate TH1 cells through the STAT3 and mTOR pathways and up-regulate the expression of Blimp-1 and IL-10, via GRP43 [46]. Acetate was also reported to promote intestinal IgA and IgA+ responses in mice through the GRP43 [47]. In addition, the acetate could induce expression of Aldh 1a2 in DCs that helps convert vitamin A to retinoic acid (RA), resulting in B- cell IgA production via GRP43. This indicates that SCFA could regulate and boost host immunity through not only their respective receptors but also in an independent manner. 

### 2.2. Production of Vitamins and Bile Acids

Along SCFA, the gut microbes also produce micronutrients including vitamins, conjugated linoleic acid, and secondary bile acids [48], and many unidentified natural products [49], which all appear to have beneficial effects on the host and microbial metabolisms. In vivo metabolomics studies found hundreds of gut microbiota-derived or dependent components in the blood and tissues of host animals [50]. Gut microbes, such as *Bacteroides fragilis*, *Enterococcus faecium*, *Enterobacter agglomerans*, and *Eubacterium lentum*, produce vitamin K2, which can reduce the risk of atherosclerosis and coronary heart diseases [51,52]. The gut microbiota can also produce vitamin B (B5 and B12) that are important for the functions of the nervous system, and their deficiency often associated with neurologic dysfunction, psychiatric and gastrointestinal, malabsorption, and insomnia disorders [53,54]. The gut microbiota also plays a vital role in the metabolism of bile acids to secondary bile acids, which include deoxycholic acid (DCA), hyodeoxycholic acid (HDCA), and lithocholic acid (LCA). The gut microbiota regulates not only the metabolism of secondary bile acids but can also inhibit the production of bile acid in the liver by reducing the inhibition of FXR in the ileum of mice [55]. In *Clostridioides difficile*-infected patients, higher levels of primary bile acids and lower levels of secondary bile acids were observed, but these levels could be restored by fecal microbiota transplantation from a healthy donor [56]. The activation of nuclear receptor farnesoid X receptor (FXR) and pregnane X receptor (PXR) by bile acid regulates the glucose metabolism, haptic autophagy, and bile acid synthesis in the ileum and liver of the host [57,58]. The secondary bile acids (LCA and acetylated DCA) are the main ligands for the PXR. In contrast, conjugated acids do not activate PXR, which can be expressed in the liver and intestinal tissues [59]. Secondary bile acids also act as potent activators of membrane G-protein coupled receptor (TGR5). The TGR5 expression has been identified in the liver, spinal cord, and astrocytes, immune cells, spleen as well as other organs. TGR5 activation by bile acids appears to regulate macrophage, adenylate cyclase, glucose metabolism, accumulation of intracellular cyclic AMP, and calcium mobilization [60]. In addition, several studies have reported that TGR5 activation regulates GLP-1, postprandial insulin release, and blood glucose levels in intestinal entero-endocrine L cells [61]. In addition, it provokes the release of glucose-induced insulin via cyclic AMP and the calcium-dependent pathway in pancreatic beta cells [62]. Secondary bile acids were also found to have an antibacterial function, limiting the growth of bile acid-intolerant bacteria by disturbing the bacterial cell membrane integrity and their intracellular contents [58]. Gut-residing bacteria have also been shown to suppress the synthesis of tauro-β-muricholic acid (T-βMCA) in the liver by alleviating FXR inhibition in the ileum [55]. T-βMCA is a primary bile acid produced by the liver and it acts as a ligand for FXR in the intestine. FXR expression in the intestine regulates not only intestinal fibroblast growth factor 15 (FGF15/19) expression but also regulates hepatic cholesterol 7a-hydroxylase (CYP7A1) expression in vivo [55,63].

### 2.3. Production of Microbial Cellular Components

The gut microbiota affects or improves the host health not only through the production of metabolic products but also by the production of cellular components, such as lipopolysaccharides (LPS), peptidoglycan, lipoteichoic acid (LTA), flagellin, and DNA. These act as ligands for pattern recognized receptors (PPRs), including the toll-like receptors (TLRs) expressed by most immune cell types, including IECs. To date, 10 types of TLRs in humans and 13 types in mice have been identified and each TLR has a separate specificity to recognize MAMPs from the microbes. For example, TLR2 serves as a receptor for Gram-positive bacteria cellular components [64], while TLR4 is a receptor for Gram-negative bacteria cell wall components, such as LPS [65]. TLR3 is a receptor for the dsRNA of most viruses [66], and TLR9 responds to unmethylated cytidine-phosphate guanosine DNA motifs, which are presented abundantly in the bacteria [67]. Microbiota-mediated TLRs signaling is required for maintaining the intestinal homeostasis and healing intestinal injury [68]. The gut commensal bacteria also suppress the inflammatory response and promote immunological tolerance, mainly through interactions with TLRs [69]. Antibiotic treatments increase the severity of colitis in mice by depleting the gut microbiota and their cellular components that mediate the signals through interactions with the TLRs to maintain the gut homeostasis and improve tissue repair [68]. The Bacteroides fragilis cellular component polysaccharide A (PSA) acts as a ligand for TLR2, which exhibits an anti-inflammatory status by activating DCs, Treg cells, increasing IL-10 production from clonal CD4+ T cells, and suppressing the production of Th17 cells in the intestine [70,71,72]. The dsRNA of commensal lactic acid bacteria (LAB) interacts with TLR3 and induces the production of protective IFN-β, which ameliorates mice from experimental colitis [73]. Microbe-derived flagellin signaling through TLR5, which is expressed on DCs in the lamina propria, maintains the level of RegIII-γ [74]. Gut microbiota-related TLRs signaling is also very important for maintaining and improving the intestinal homeostasis and repair after an intestinal injury [75]. 

IECs express TLRs and nucleotide-binding oligomerization domain-containing protein (NOD)-like receptors that can recognize the gut microbiota-derived structural components and regulate antimicrobial compounds production by Paneth cells [76,77]. Meso-diaminopimelic acid (DAP), a cell wall component of gut bacteria, is sensed by NOD-1 and mediates signaling, which then induces neutrophils to kill pathogenic bacteria, such as *Streptococcus pneumonia* and *Staphylococcus aureus* [78]. Antigen-presenting cells (APCs) and T cells can also detect the gut microbiota and the crosstalk between APCs and T cells link the innate and adaptive immune system in humans and animals. The gut microbiota, especially segmented filamentous bacteria (SFB), regulates the Th17 response in the gut of mice [79]. Intestinal monocyte-derived macrophages control the SPB-specific Th17 cells responses [80]. Gut bacteria *B. fragilis* is a potent bacterium that activates DCs via TLR2 to induce the differentiation of inducible Treg cells and the production of IL-10 [71,81]. Commensal flagellin induces lamina propria DCs (CD172α+ LPDCs) cells to promote Th17 cell development and produce higher levels of IL-6, IL-23, and TGF-β through an interaction with TLR5 [82]. In addition, innate lymphoid cells (RORγt+) can regulate CD4+ T cells via major histocompatibility complex class II (MHCII) and limit the pathological responses to commensal microbiota [83].

SFB colonization in mice activates innate lymphoid cells (ILC3) to secrete IL-22 that induces serum amyloid A protein 1 and 2, production to promote local IL-17A responses [84]. Commensal *Clostridia* strains protect mice from experimental colitis by elevating the production of TGF-β and increasing the accumulation of Treg cells in the colon of mice [85]. The gut microbiota and their cellular components are also important for the development of B cells. Microbiota-driven IL-6 and IL-1β promote regulatory B cells (Breg) in the spleen and the mesenteric lymph nodes in mice [86]. Moreover, the LPS of commensal bacteria are important for the development of B cells in the spleen of mice and for maintaining the circulating IgM level in mice [87]. The gut microbiota can also confer protection against the colonization of pathogenic microbes by colonization resistance. This takes place by two mechanisms: direct interaction of the microbiota with pathogens competing for nutrients and the indirect induction of the host defense system, such as the production of antimicrobial peptides (AMPs), IgA, or anti-inflammatory cytokines to suppress pathogenic invaders [88,89]. Commensals *Blautia producta* and *B. thetaiothaomicron* exhibit resistance to *Candida albicans* by activating the expression of HIF1-α and increasing the production of antimicrobial peptides (LL-37) in mice [90]. During dysbiosis, however, the opportunistic pathogens or pathobionts (*Clostridium difficile*) evolve to occupy empty niches, and result in the development of bacterial infections or diseases [91]. Overall, the gut microbiota plays a crucial role in improving host health by revealing direct or indirect signals to the sensors or receptors of the host epithelial and immune cells.

## 3. Gut Microbiota and Diseases

Many factors, such as dietary products, antibiotic treatment, inflammatory products (LPS, flagella, LTA), and host physiological stress, have been shown to induce dysbiosis in gut microbiota composition. Dysbiosis impairs the functions of microbes and selectively influences the growth of pathobionts, which dysregulate the production of microbial products that induce the development of several diseases on local or neighbor organs, such as inflammatory bowel diseases (IBD), *Clostridium difficile* infection (CDI), obesity, metabolic syndrome, and diabetes. The decreased abundance of enteric bacterial diversity and the increased richness of *Enterobacteriaceae* have been strongly correlated with IBD [92,93], and the depletion of *Enterobacteriaceae* by the tungstate treatment ameliorates the severity of intestinal inflammation in mice with colitis [94]. The relatively higher abundance of *Enterobacteriaceae* and *B. fragilis* increased the levels of LPS that induce intestinal inflammation and colitis in mice through the suppression of Treg cells and the activation of T_H_1, and T_H_17 cells [95]. *Faecalibacterium prausnitzii*, an anti-inflammatory commensal bacterium, and its loss induces the reoccurrence of Crohn disease (CD), while its administration reduces inflammation by increasing anti-inflammatory cytokine (IL-10) and decreasing inflammatory cytokines (IL-12, IFN-γ) in a mouse model of colitis [96]. *Clostridium difficile* is a normal bacterial member in the gut, but it is also a pathobiont that reduces the intestinal epithelial cell integrity and induces inflammatory activity and cell death [97]. *Clostridium difficile* infection is often associated with antibiotic-mediated diarrhea that can be resolved by the dietary carbohydrate reduction [98]. 

Early findings indicated that the gut microbiota plays a role in the development of obesity in humans and animals. A lower diversity of gut microbes was observed in overweight and obese people [99]. Germ-free mice that received microbiota from obese subjects were heavier than the mice that received microbiota from healthy humans [100]. Perturbations in the composition of gut microbiota promote diet-induced obesity and metabolic diseases through different mechanisms, such as immune dysregulation, increased LPS production, altered energy, and gut hormone regulation [101]. The diet can also induce perturbations to the composition of gut microbiota and their functions. A study reported that mice fed a high-calorie Western diet showed a low number of Bacteroidetes and a high number of Firmicutes [102]. The high intake of sweeteners by humans or mice significantly altered the gut microbiota composition and induced glucose intolerance [103]. Rats fed sucralose exhibited an increased abundance of *Bacteroides*, *Clostridia*, and anaerobic bacteria in the gut [104]. Therefore, changes or loss of gut microbiota are associated with dysbiosis and are capable of inducing intestinal inflammation and other disorders that can be restored or ameliorated by supplementation with probiotics or prebiotics/dietary fibers [105,106]. In addition, studies suggest that alterations of the gut microbial composition extend its effects beyond the digestive system and can affect the functions of extra-intestinal organs, such as the liver. Figure 1 outlines how the gut microbiota contributes to pathogenic diseases and the mechanisms underlying the healthy and pathogenic state. This study discusses how the gut microbiota affects the liver function and promotes hepatic diseases via the gut–liver axis.

## 4. Gut–Liver Axis in Liver Diseases

Anatomically, the liver has a strong relationship with the gut where the gut microbes and their metabolites, nutrients, and gut-derived hormones contribute to the maintenance of healthy liver and liver metabolisms. The liver is not only a receiver of gut-derived products, but it also responds to the intestine by producing bile acids and IgA that affect the gut–liver axis [107]. Interestingly, liver-derived bile acid has been reported to induce changes in the gut microbiota composition [5]. Human and mouse liver contain gut-derived IgA-secreting cells that could mediate clearance of gut-derived antigens and protect the liver from pathogens, which reflects the strong connection between the gut and liver [108]. On the other hand, the bidirectional relationship between the gut and liver is normal in the healthy state, but during gut dysbiosis, a large number of gut bacteria and their derivatives, and microbial metabolites translocate to the liver, primarily through the portal vein, resulting in liver injury and the progression of liver diseases. The stage of liver disease truly relies on the severity of gut dysbiosis.

Studies have suggested the involvement of the gut microbiota in the development of non-alcoholic fatty liver disease (NAFLD), which is one of the most life-threatening liver diseases worldwide. Dysbiosis of the gut microbiota in the intestine of humans is associated with inflammation and impairments in mucosal immune function, which play a vital role in the pathogenesis of NAFLD [109]. Germ-free mice fed a high-fat diet (HFD) showed lower levels of lipids in the liver compared to HFD fed conventional mice [110]. In addition, germ-free mice received gut microbiota from hyperglycemia and insulinemia mice showed the development of NAFLD compared to the mice that received microbiota from normal mice [111]. Two bacterial species, *Lachnospiraceae bacterium* 609 and *Barnesiella intestinihominis*, were reported to be higher in the stool samples capable of inducing NAFLD, while *B. vulgatus* was found to be lower compared to the control group [111]. An increased abundance of *Escherichia*, *Lactobacillus*, *Anaerobacter*, and *Streptococcus* spp. was observed in NAFLD patients compared to healthy subjects [109]. Moreover, higher levels of TNF-α, IL-6, and IFN-γ, and a lower number of CD4+ and CD8+ cells were detected in NAFLD patients [109]. NAFLD can also develop into non-alcoholic steatohepatitis (NASH), which turns into fibrosis, cirrhosis, and hepatocellular carcinoma (HCC). Pathogen-free mice that received gut microbiota from HFD-fed mice showed gut vascular barrier (GVB) disruption and epidydimal adipose tissue enlargement through interference with the WNT/β-catenin signaling pathway in endothelial cells [2]. The increased GVB allows gut bacterial translocation to the liver, where they activate the parenchymal and non-parenchymal liver cells via pattern recognized receptors and others to develop various liver diseases [2,112]. Induction of intestinal inflammation increases gut-microbe-derived LPS in the portal circulation, resulting in hepatic inflammation and liver fibrosis, with the increased expression of collagen1, TIMP-1, TGF-β, PAI-1, and α- smooth muscle actin (SMA) in an experimental NASH mice model [113]. Liver fibrosis is a next stage of NASH. It can be induced by liver resident cells, especially hepatocytes, Kupffer cells (KCs), and hepatic stellate cells (HSCs). These cell types can express TLRs that are capable of recognizing microbe-derived ligands and mediate dangerous signals, resulting in liver injury. 

### 4.1. Role of TLRs/Nod-Like Receptors Signaling on Liver Disease

Among the TLRs, TLR4 is a receptor for LPS that translocates through the leaky gut [3]. Myeloid differentiation factor 88 (MyD88) is a common adaptor molecule that transmits inflammatory signaling of TLR4 by recruiting a series of proteins (tumor necrosis factor receptor-associated kinases 3, 6 (TRAF3 and 6), IL-1 receptor-associated kinases (IRAKs), TGF-β activated kinase 1 (TAK1), TAK1-binding protein 2 (TAB2)), activating nuclear factor (NF)-kappa B (NF-κB), mitogen-activated proteins kinase (MAPKs), and interferon regulatory factors (IRFs) pathways and inducing the production of inflammatory cytokines, such as IL-1β, TGF-β, TNF-α, and IL-6 [114,115]. The primary KC cells respond to LPS via expression of TLR4, but the responsiveness of KCs to LPS is much lower. This is probably due to their LPS tolerance [116]. On the other hand, in response to LPS, the cells increase the expression of pro-inflammatory cytokines (IL-1β, IL-12, and IL-18), which induces natural killer (NK) cells and cytotoxic T cells [117,118]. HSCs are the prominent cells that produce higher levels of extracellular matrix proteins to induce liver fibrosis [3]. HSCs respond strongly to LPS and activate TGF-β signaling to induce liver fibrosis by decreasing the TGF-β pseudoreceptor and activin membrane-bound inhibitor (Bambi) proteins and increasing cytokine/chemokine (CCL2, CCL3, CCL4, and CCL5) and adhesion molecule (ICAM-1, Coll1A, and VCAM-1) expressions [3]. Several studies reported that a deficiency of TLR4, MyD88, and myeloid differentiation factor-2 (MD-2) expression attenuate NASH and liver fibrosis in mice treated with carbon tetrachloride (CCl4) and a methicillin choline-deficient diet (MCD) [3,119]. Sterilization of the gut resulted in a lower level of plasma LPS resulting in the amelioration of liver fibrosis in mice, suggesting that bacteria-derived LPS play a role in liver fibrosis [3]. In addition, TLR9 is also involved in the development of liver fibrosis and its deficiency suppressed the progression of liver fibrosis in mice treated with CCl_4_ [120]. The activation of TLR4 signaling by LPS suppresses the expression of micro RNA (miR-29a and miR-29b, a family of small non-coding RNAs can control the translation of many genes) in HSC cells, leading to the activation of HSC cells and liver fibrogenesis [121]. The increased miR-29 expression could counteract collagen expression in murine HSC cells [121]. In addition, the miR-101 family, a suppressor of TGF-β signaling, was lower in the CCl_4_-induced liver fibrosis mice model. The higher level of miR-101 inhibited TGF-β signaling through the suppression of TβR1 expression in both hepatocytes and HSC cells [122]. On the other hand, the overexpression of miR-155 promotes TLR4/LPS signaling, resulting in an increase in alcohol-induced TNF-α production in KCs through the stabilization of higher levels of mRNA [123]. Similarly, KCs isolated from alcohol-fed mice exhibited a lower level of IRAK-M, SHIP1, and PU.1, and a higher level of TNF-α. In contrast, KCs isolated from miR-115 KO mice after the LPS treatment reversed the expression of these genes [124]. In addition, miR-155 overexpression decreased the level of IRAK-M, SHIP1, C/EBPβ, IL-10 in LPS, and alcohol-treated KCs. A knockdown of HDAC11 (a regulator of IL-10) increased IL-10 in alcohol and LPS pretreated macrophages. Overall, alcohol-induced miR-155 and HDAC11 restrain the TLR4 negative regulators by increasing the responsiveness of KCs to LPS in alcohol liver diseases [124]. 

TLR9 signaling activated KCs and increased the production of IL-1β, which resulted in the induction of steatohepatitis and fibrosis in mice [125]. A lack of TLR9 reduced CDAA-induced steatohepatitis and fibrosis in mice [125]. Human HSC cells and HSC cells from TLR9-deficient mice expressed a higher level of MCP-1, in response to CpG motif in vitro. In addition, bile duct ligation (BDL) showed significantly lower levels of hepatic MCP-1, collagen deposition and fibrosis in TLR9-deficient mice [120]. TLR2 has a protective role against MCD diet-induced NASH [126], but one study reported that a TLR2 deficiency suppressed the development of NASH in mice treated with choline-deficient amino acid (CDAA) [127]. Furthermore, inflammasome (NLRP-3, NLRP-6), a member of the NOD-like receptors family, plays a role in the microbiota-mediated induction of NASH [128]. The expression of NLRP-1 and NLRP-3 was higher in rats treated with LPS, CCl_4_, and subjected to bile duct ligation (BDL); this was also observed in KCs and HSCs [129]. Rats with cirrhosis exhibit gut dysbiosis, which impairs intestinal immune dysregulation, leading to gut barrier disruption and gut bacterial translocation [130]. The number of intestinal T helper cells (CD3+CD4+), cytotoxic T cells, NK cells, and TNF-α were increased in rats with cirrhosis, while the number of Th17 cells were decreased [130]. Patients with cirrhosis exhibited elevated levels of serum LPS [131]. At the same time, the expression of TLR4 was lower in the PBMC of cirrhosis patients after the antibiotic treatment, indicating the systemic hyporesponsiveness of LPS to TLR4 in patients with cirrhosis [132]. In addition, elevated levels of bacterial DNA were observed in the circulation and ascitic fluid of cirrhotic patients, which resulted in higher levels of inflammatory cytokines (IL-16, IL-12, iNOS, and TNF-α) in the plasma of patients [133]. The stimulation of HSC cells with LPS induced the progression of HCC through the activation of protein kinase R (PKR) [134]. A recent study showed that TLR4/LPS signaling induced the differentiation of hepatic progenitor cells (HPCs) into myofibroblasts and increased the levels of IL-6 and TNF-α by activating the signaling of the hedgehog [135], Ras, and p53 pathways [4]. HPC-mediated myofibroblasts play a vital role in the proliferation and transformation of HPCs into the malignant form [4]. The liver is the primary secretor of BA that can be modified into secondary BA by the gut microbiota. The presence of BA activates FXR and TGR5, which ameliorate hepatic inflammation and hepatic steatosis in vivo [136]. In addition, primary BA increased the accumulation of hepatic NKT cells and reduced tumor growth by increasing CXCL16 expression in mice with HCC. In contrast, secondary BA reversed the results [137]. Recently, the triggering receptor expressed in myeloid cells 1 (TREM-1) signaling plays a role in liver injury and hepatic fibrosis. Moreover, its expression is not limited in hepatic macrophages and is expressed strongly in KC cells derived from mice with chronic liver injury and liver fibrosis [138]. A deletion of TREM-1 could attenuate liver injury, as well as the recruitment and differentiation of inflammatory cells, and liver fibrosis through the reduction of TGF-β, α-SMA, Col1a1, Col5a1, Acta2, MMP10, and Birc5. The activation of TREM-1 in KC induces quiescent HSC cells and activates fibrogenic HSCs via the production of TGF-β [138]. Oxidative stress or ROS production plays a major role in the development of NAFLD and NASH [139,140]. Activation of Na/K-ATPase/ROS signaling may stimulate macrophages to produce higher levels of pro-inflammatory cytokines/chemokines (TNF-a, IL-18, IL-6, and MCP-1) that promote the development of NAFLD and NASH [139,141]. The production of cytokines/chemokines also activates KC cells, which in turn, induce the lipid metabolism and storage in hepatocytes [142,143].

### 4.2. Role of Gut Microbiota on Alcoholic and Viral-Induced Liver Diseases

The gut bacteria also play a key role in the severity of alcoholic liver diseases (ALD), hepatitis B virus (HBV), hepatitis C virus (HCV) infection-induced liver fibrosis, and HCC. The long-term consumption of excessive alcohol results in ALD. Alcohol and its metabolites disrupt the gut barrier and increase gut permeability and intestinal inflammation [144]. The increased gut permeability results in higher translocation of gut-derived endotoxin (LPS) [145], which is capable of activating KCs and recruiting macrophages by interacting with TLR4, which leads to liver injury [146]. The elevated serum LPS level was observed in patients and mice with chronic alcohol consumption or administration [147,148]. TLR4 signaling is required for bone marrow (BM)-derived KCs and endogenous HSCs in alcohol-induced liver steatosis and fibrogenesis in mice [149]. TLR4 chimeric mice and TLR4(^−/−^) mice transplanted with TLR4(^−/−^) BM exhibit lower expression levels of fibrogenic markers (Colα1(I), TIMP1, TGF-β1), and inflammatory cytokines (IL-1β and IL-6) than the wild type (WT) mice with WT BM that were treated with intragastric alcohol administration [149]. Mice fed alcohol showed increased intestinal bacterial overgrowth with a relative abundance of *Bacteroidetes* and *Verrucomicrobia* bacteria and lower expression levels of bactericidal c-type lectins Reg3b and Reg3g in the small intestine [150]. A recent study reported that chronic ethanol feeding increases Gram-negative *Prevotella*, a source of endotoxins, in the mucus later of ileum and the liver samples of mice [151]. In addition, ethanol exposure decreases the abundance of intestinal *Akkermansia muciniphila* in both humans and mice, resulting in increased hepatic injury, steatosis, and neutrophil infiltration, which could be restored by the supplementation of *A. muciniphila* in mice [152]. Alcohol consumption is also associated with increases in the abundance of endotoxin-producing *Enterobacteriaceae* and decreases in the abundance of SCFAs-producing *Lachnospiraceae* and *Ruminococcaceae* [1,153]. Furthermore, the composition of gut microbiota differed according to the level of alanine aminotransferase (ALT) in HBV patients [154]. *Desulfovibrio* had a positive correlation, while *Acidaminococcus* showed a negative correlation with a high level of ALT [154]. Chen and coworkers analyzed the changes of gut bacterial composition in HBV related cirrhotic patients. The level of *Firmicutes*, *Veillonella*, *Megasphaera*, *Dialister*, *Atopobium* and *Prevotella* were found to be higher in cirrhotic duodenum, whereas the duodenum of healthy controls was enriched with *Haemophilus*, *Neisseria* and *SR1 genera incertae sedis* [155]. Xu et al. [156] characterized the composition of intestinal *Bifidobacterium* in patients with HBV-induced chronic liver disease. Authors reported that the composition of *Bifidobacterium* was significantly altered in HBV patients with a shift from beneficial to opportunistic pathogens. The lower level of phylum *Bacteroidetes* and the higher levels of *Firmicutes*, *Proteobacteria* and *Actinobacteria* were found in patients with hepatic encephalopathy (HE), which is a serious complication in viral hepatitis cirrhosis [157]. In addition, the abundance of pathogenic bacteria *Enterobacteriaceae*, *Enterococcus* and *Staphylococcus* were found to be higher in HCV patients. These increased levels of bacteria were decreased when patients were treated with antiviral therapy [158]. In addition, alternation in gut microbiota composition was observed in patients with HCV [159]. Authors suggest that HCV infection is correlated with a lower level of alpha diversity and different microbial community patterns. The interactions between microbiota and HCV might be facilitated by the immune system [159].

**Figure 2:**
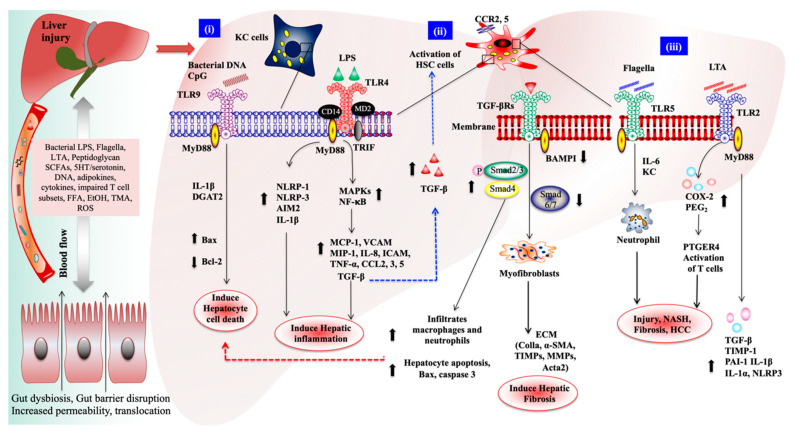
Schematic diagram of gut bacteria-derived components/metabolites on the promotion of liver diseases via the interactions with TLRs signaling. Chronic alcohol abuse and liver diseases induce gut dysbiosis, increase the gut permeability, bacterial overgrowth, systemic inflammation, resulting in the translocation of microbial components (lipopolysaccharides (LPS), lipoteichoic acid (LTA), flagella, and DNA), metabolites (short-chain fatty acids (SCFAs), trimethylamine (TMA), free fatty acids (FFAs), and ethanol (EtOH)), inflammatory cytokines, and impaired T cells to the liver via the portal and lymphatic circulation, where they mediate inflammatory signaling that has been shown to promote the development of non-alcoholic steatohepatitis (NASH), fibrosis, and hepatocellular carcinoma (HCC). (i) The interaction of bacterial DNA or CpG-DNA with TLR9 activates Kupffer cells (KCs) and increases the production of IL-1β, which binds with IL-1R on hepatocytes, induces intracellular fat accumulation, and nuclear factor (NF)-kappa B (NF-κB) activation, which promotes hepatic steatosis and cell death by increasing the level of Bax and decreasing the level of Bcl-2. The translocation of LPS activates TLR4/MyD88 signaling on both Kupffer (KC) and hepatic stellate cells (HSCs), and recruits IRAK, TRAF6, TAIK, and TAB to activate the mitogen-activated proteins kinase (MAPK) and NF-κB pathways, resulting in the up-regulation of cytokines/chemokines production, including pro-fibrogenic transforming growth factor-β (TGF-β). LPS also activates KC cells and induces the production of NLRP1/3 inflammasomes, AIM2, and IL-1β, which contributes to the progression of liver fibrogenesis and HCC. (ii) KC and HSC cell-produced TGF-β stimulate fibrogenic transforming growth factor-β receptor 1 and 2 (TGF-βR1&2) signaling on HSC cells and induce liver fibrogenesis by up-regulating the Smad2/3/4 proteins and down-regulating bone morphogenic protein and activin membrane-bound inhibitor (BAMBI) (TGF-β receptor inhibitor) and Smad6/7. TGF-β/Samd3 signaling infiltrates more neutrophils and macrophages to the liver and increases hepatocyte apoptosis and the related protein expression, such as Bax cytochrome and cleaved caspase 3. The activation of HSC cells can recruit and stimulate hepatocytes, macrophages, and KC via paracrine signaling using TGF-β and connective tissue growth factor (CTGF). (iii) The translocation of lipoteichoic acid (LTA) and flagella can also promote liver injury, NASH, fibrosis, and HCC via the activation of respective TLRs (TLR2 and TLR5) signaling on KC and HSC cells [3,113,119,120,121,125,146,164,165]. Arrows (🠕🠗) indicates up and down-regulation of genes. Solid lines indicate production genes and dashed lines (blue and red) indicate further activation of HSCs and cell death.

In HCC patients and mice, changes in the composition of gut microbiota were observed with an increase in the abundance of *E. coli*, a Gram-negative bacterium associated with higher levels of serum LPS [160], and a decrease in the level of *Lactobacillus* and *Bifidobacterium* spp., and *Enterococcus* spp [161]. In HBV-related HCC patients, an altered gut microbiota composition was observed with an increased abundance of *Prevotella* and a decreased abundance of *Faecalibacterium*, *Lachnoclostridium*, *Ruminoclostrdium*, *Pseudobutyrivibrio*, and *Phascolarctobacterium*, which are potent bacterial spp. that improve the anti-inflammatory activity of SCFAs, particularly butyrate [162]. One study also reported that intestinal bacteria and the activation of TLR4 signaling promote the development of HCC by mediating the increased cell proliferation, expression of hepatomitogen epiregulin, and suppression of apoptosis [163]. Intestinal dysbacteriosis-induced IL-25 promotes the development of HCC through alternative activation and the secretion of CXCL-10 by macrophages in the tumor microenvironment [164]. Furthermore, depletion of the gut microbiota by an antibiotic treatment inhibited HCC development in mice by increasing the accumulation of hepatic NKT cells and effector memory CD4+ or CD8+ T cells [137]. The higher levels of primary BA, CXCL16, and CXCR6 could regulate the accumulation of hepatic NKT cells in HCC mice. LTA is a gut bacterial component that acts as a ligand for TLR2, promoting HCC development in obese mice by increasing the senescence-associated secretory phenotype (SASP) of HSC and COX2 expression. The COX2-induced prostaglandin E2 (PGE2) counteracts the antitumor immunity via the PTGER4 receptor, which leads to the progression of HCC. Moreover, the levels of both COX2 and PGE2 were higher in HSCs and noncirrhotic and NASH-associated human HCC [165]. The gut-derived metabolite indole-3-acetic acid (IAA) alleviates high-fat diet-induced hepatotoxicity via the amelioration of hepatic lipogenesis, lipid metabolisms, and oxidative and inflammatory stress in mice [166]. Figure 2 outlines the gut microbiota and their contribution to the progression of liver diseases, and the response of hepatic cells to gut-derived components and the related mechanisms. 

Abbreviations used in the figure are: Bone morphogenic protein and activin membrane-bound inhibitor (BAMBI), transforming growth factor-β receptor 1 and 2 (TGF-βR1&2), chemokine receptors (CCR2, 3, 5), diacylglycerol acyltransferase 2 (DGAT2), plasminogen activator inhibitor-1(PAI-1), tissue inhibitor of metalloproteinase-1 (TIMP1), prostaglandin E2 (PGE2), prostaglandin E receptor 4 (PTGER4), lipoteichoic acid (LTA), free fatty acids (FFA), ethanol (EtOH), keratinocyte chemoattractant (KC), cyclooxygenase 2 (COX2), NOD-like receptor family pyrin domain-containing 1 and 3 (NLRP1 & 3), α-smooth muscle actin (α-SMA), connective tissue growth factor (CTGF).

## 5. Therapeutic Approaches

Several therapeutic approaches have been shown to restore the changes in gut microbiota composition and improve liver diseases, which include antibiotics treatment, prebiotics, probiotics supplementation, and fecal microbiota transplantation. Oral administration of antibiotics improved liver fibrosis [4] and alcohol-induced steatohepatitis [62]. On the other hand, a long-term approach may cause microbiome changes in the intestine of animals and humans. Probiotics are beneficial live microorganisms that have been shown to prevent liver diseases by reducing bacterial translocation and their derivatives and maintaining the gut barrier integrity [167,168,169,170]. *L. rhamnosus* R001 and *L. acidophilus* R0052 could ameliorate ALD in mice by reducing TLR4 expression and inflammatory cytokine (IL-6, IL-10, and TNF-α) production in mice [171]. Another study also showed that the development of ALD was prevented in mice treated with *L. rhamnosus* GG (LGG) by positively modulating bacterial composition to stimulate long-chain fatty acids (LCFAs) production, and increasing amino acid concentration in the intestine and liver of mice [172]. A combination of blueberry and probiotics has been reported to ameliorate NASH by increasing the level of peroxisome proliferator-activated receptor α (PPAR-α) and decreasing the level of sterol regulatory element-binding protein-1c (SREBP-1c), patatin-like phospholipase domain-containing protein 3 (PNPLA-3), and inflammatory cytokines (TNF-α, IL-6), Bcl-2 and caspase-3 in mice with NASH [173]. Clinical trials also confirmed the beneficial activity of probiotics against liver diseases, such as NAFLD/NASH and ALD in patients [174,175]. Prebiotics (inulin and pectin) are barely digestible food ingredients that have been reported to prevent hepatic injury, hepatic lipogenesis, and plasma triacylglycerol concentration by restoring Bacteroides in mice [176] and increasing SCFAs in humans [177]. Collectively, these studies provide evidence for the therapeutic potential of pre/probiotics on the amelioration of liver diseases, including NAFLD/NASH, ALD, and its related cirrhosis. There were no negative or adverse effects reported in clinical trials and mouse models using pre/probiotics. 

Moreover, fecal microbiota transplantation (FMT) has attracted more attention for its ability to restore the gut microbiota and improve liver diseases. Alcohol-sensitive mice received FMT from alcohol-resistant donor mice that prevented the severity of ALD-induced liver injury, inflammation, steatosis and gut dysbiosis in vivo [176]. FMT improved rat behavior, hepatic encephalopathy (HE) grade and spatial learning capability in rat. In addition, FMT reduced intestinal permeability, intestinal mucosal barrier damage and systemic inflammation in rat treated with CCl_4_ [173]. Moreover, FMT in rat restored HE-induced losses of Claudin-1, Claudin-6 and Occludin in intestinal tissues of rat [178]. The liver disease indices were significantly improved within the first week after FMT therapy in patients with alcoholic liver disease [179]. Furthermore, FMT restored the gut microbiota composition, improved the metabolic pathways, bile secretion, carotenoid and pantothenate biosynthesis to normal levels in alcoholic liver disease patients. In a translational study, FMT from patients with alcoholic hepatitis induced severe inflammation, hepatic necrosis, intestinal permeability and bacterial translocation in germ-free humanized mice [180]. In addition, mice received from patients without alcoholic liver disease improved lesions in the liver, confirming the therapeutic roles of gut microbiota in alcoholic hepatitis. 

In addition, herbal medicine (Sha-saiko-to) has potent effects and is used widely to treat patients with liver cirrhosis and HCC [181]. Herbal medicine has been shown to inhibit the activation of HSC cells, reduce hepatic lipid peroxidation, accumulation of extracellular matrix (ECM), expression of tissue inhibitor of metalloproteinases (TIMPs), and increase MMPs in rats [182,183]. A recent study reported that a Chinese herbal medicine (extract of *Graptopetalum paragusyense*) ameliorated dimethylnitrosamine (DMN)-induced hepatic inflammation and hepatic fibrosis via the suppression of TGF-β signaling in rats and rat HSC cells [184]. Overall, knowledge of the gut–liver axis has increased over the last decade through a series of microbiome studies that confirmed the vital role of the gut microbiota in chronic liver diseases.

## 6. Conclusions

The gut–liver axis plays a vital role in the etiology and pathogenesis of liver diseases. Gut-derived metabolites, cellular components, hormones, and others are translocated to the liver via the portal circulation, where they actively interact with immune cells and perform an inflammatory response, as well as induce the progression of several liver diseases. Among the gut-derived components, LPS is a key inflammatory molecule, which increasingly translocates to the liver during gut dysbiosis. Therefore, the TLR4/LPS signaling pathway is actively involved in the pathogenesis of liver diseases. In contrast, gut-derived SCFAs and BAs have beneficial effects that improve the liver functions. Current clinical and animal trials with different therapeutic strategies improve the present knowledge of the gut–liver axis, showing a favorable result that gives future hope to combat and ameliorate liver diseases.
